# Pro-inflammatory cytokines in cystic glioblastoma: A quantitative study with a comparison with bacterial brain abscesses. With an MRI investigation of displacement and destruction of the brain tissue surrounding a glioblastoma

**DOI:** 10.3389/fonc.2022.846674

**Published:** 2022-07-29

**Authors:** Bjørnar Hassel, Pitt Niehusmann, Bente Halvorsen, Daniel Dahlberg

**Affiliations:** ^1^ Department of Neurohabilitation, Oslo University Hospital, Oslo, Norway; ^2^ Institute of Clinical Medicine, University of Oslo, Oslo, Norway; ^3^ Norwegian Defence Research Establishment (FFI), Kjeller, Norway; ^4^ Department of Pathology, Oslo University Hospital, Oslo, Norway; ^5^ Division of Cancer Medicine, Oslo University Hospital, Oslo, Norway; ^6^ Research Institute of Internal Medicine, Oslo University Hospital, Oslo, Norway; ^7^ Department of Neurosurgery, Oslo University Hospital, Oslo, Norway

**Keywords:** glioblastoma, macrophage, cytokine, tumor microenvironment, brain abscess, pus, inflammation, cyst fluid

## Abstract

Cystic glioblastomas are aggressive primary brain tumors that may both destroy and displace the surrounding brain tissue as they grow. The mechanisms underlying these tumors’ destructive effect could include exposure of brain tissue to tumor-derived cytokines, but quantitative cytokine data are lacking. Here, we provide quantitative data on leukocyte markers and cytokines in the cyst fluid from 21 cystic glioblastomas, which we compare to values in 13 brain abscess pus samples. The concentration of macrophage/microglia markers sCD163 and MCP-1 was higher in glioblastoma cyst fluid than in brain abscess pus; lymphocyte marker sCD25 was similar in cyst fluid and pus, whereas neutrophil marker myeloperoxidase was higher in pus. Median cytokine levels in glioblastoma cyst fluid were high (pg/mL): TNF-α: 32, IL-6: 1064, IL-8: 23585, tissue factor: 28, the chemokine CXCL1: 639. These values were not significantly different from values in pus, pointing to a highly pro-inflammatory glioblastoma environment. In contrast, levels of IFN-γ, IL-1β, IL-2, IL-4, IL-10, IL-12, and IL-13 were higher in pus than in glioblastoma cyst fluid. Based on the quantitative data, we show for the first time that the concentrations of cytokines in glioblastoma cyst fluid correlate with blood leukocyte levels, suggesting an important interaction between glioblastomas and the circulation. Preoperative MRI of the cystic glioblastomas confirmed both destruction and displacement of brain tissue, but none of the cytokine levels correlated with degree of brain tissue displacement or peri-tumoral edema, as could be assessed by MRI. We conclude that cystic glioblastomas are highly pro-inflammatory environments that interact with the circulation and that they both displace and destroy brain tissue. These observations point to the need for neuroprotective strategies in glioblastoma therapy, which could include an anti-inflammatory approach.

## Introduction

Glial progenitor cells may give rise to malignant tumors ranging from the highly differentiated, low-grade astrocytomas to the undifferentiated, rapidly growing glioblastomas (1, 2); glioblastoma carries a median survival time of months only (3, 4). Because of the limitations to tumor expansion imposed by the rigid skull, it is often assumed that glioblastomas grow by destroying brain tissue (5–8) in addition to displacing it (4, 9). Indeed, several lines of evidence point to a destructive effect of glioblastomas on the surrounding brain tissue. Destruction of white matter tracts (in which glioblastomas tend to reside) has been shown with MRI-based diffusion tensor imaging (10), and loss of the neuronal marker N-acetyl-aspartate from white matter that has been infiltrated by glioblastoma has been shown with magnetic resonance spectroscopy (11). In agreement, glioblastoma patients have high circulating levels of neurofilament light chain, a marker of neuronal damage (12). Histologically, glioblastomas are seen to grow by invading the surrounding brain tissue (1, 9, 13), but the original brain tissue is hardly present within the tumor, in line with a destructive effect of glioblastomas on the brain tissue that has been invaded. A destructive or toxic effect of glioblastoma on neural cells has been replicated experimentally by several research groups (14–16). To our knowledge, no study has attempted to distinguish between destruction and displacement of brain tissue on pre-surgical MRIs of glioblastomas. This would be clinically valuable information, which may help predict functional outcome after surgery and thus guide patient information prior to surgery.

**Figure 1:**
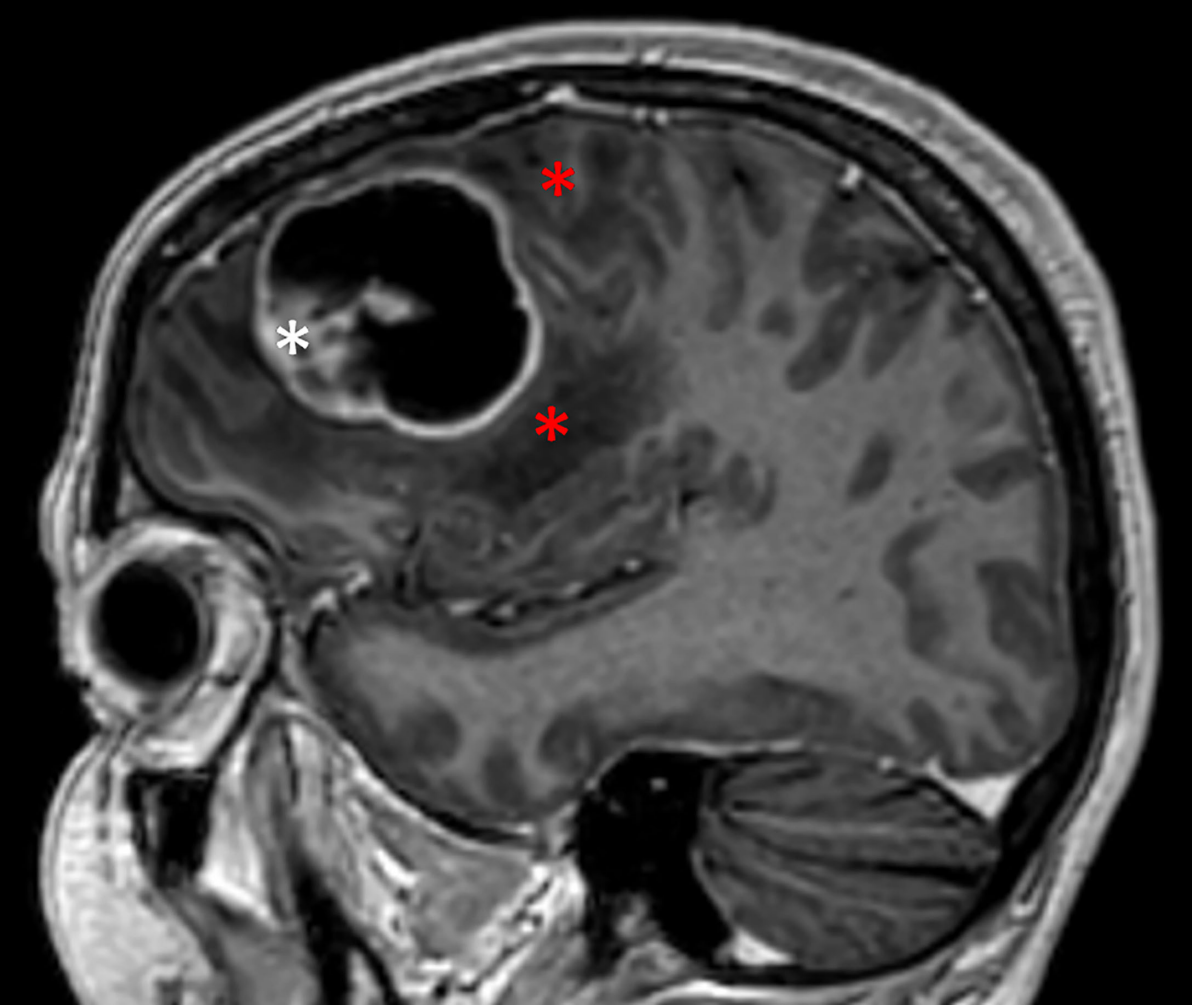
Cystic glioblastoma. Note how the cyst is in close contact with tumor tissue (white asterisk) and the surrounding brain tissue, both white matter and overlying neocortex. Red asterisks indicate the zone of peri-tumoral edema.

In principle, glioblastomas may cause destruction of normal brain tissue through a variety of processes that encompass an inflammatory response, physical strain due to the tissue distortion caused by the tumor, ischemia due to compression of vasculature or the inadequacy of the neovasculature established in the course of tumor growth, and exposure of the surrounding brain tissue to neuroactive or neurotoxic compounds. The last decades have seen the identification of several such glioblastoma-derived neuroactive compounds, including various cytokines (17, 18), glutamate (15, 19, 20), matrix metalloproteinases (21), hormones such as androgens, insulin, and erythropoietin (22–24), micro-RNAs in extracellular vesicles (25, 26), and extracellular nanotubes (27). Thus, glioblastomas may influence the surrounding brain tissue in a multi-modal fashion (25). However, the clinical importance of the various factors is not fully known, in part because of the practical difficulties of obtaining human material that allows not only the detection of such compounds, but also their quantification. One approach to solving this problem is offered by the fact that glioblastomas may have cystic compartments (28, 29). The cyst fluid is in close contact with both tumor cells and the surrounding brain tissue (**Figure 1**). Cyst fluid is aspirated during neurosurgery and lends itself to the quantitative study of the glioblastoma environment.

**Figure 2:**
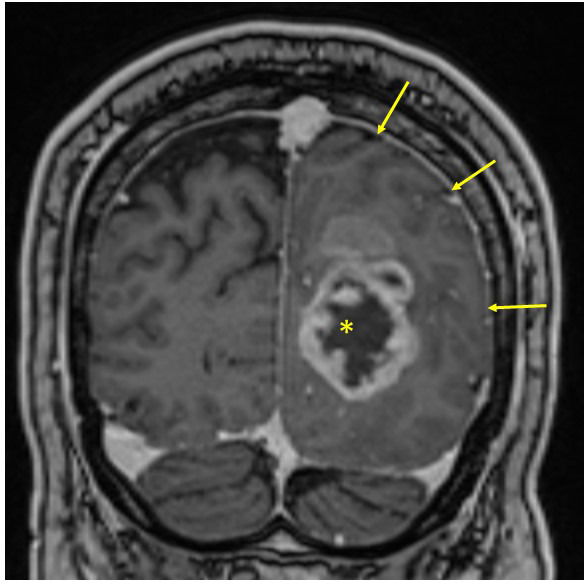
Solid glioblastoma in the left occipital lobe. Note how the tumor affects white matter (compare with contralateral side) and produces a mass effect with obliteration of the subarachnoidal spaces of the ipsilateral sulci (arrows). Centrally, this tumor has an area of necrosis (asterisk), which is not cystic.

Some aspects of the presumed neurotoxicity of glioblastomas warrant mention. First, glioblastomas tend primarily to reside in, and spread along, the white matter tracts of the brain (**Figure 2**; 30). Glioblastomas, therefore, would be expected to impact axons or their myelinating oligodendroglia, the local fibrous astrocytes, or white matter neurons (31, 32) in addition to neuronal cell bodies in the cerebral cortex. In agreement, MRI-based studies confirm a major effect of glioblastomas on white matter integrity (10, 11). Second, analysis of the cyst fluid of cystic glioblastomas has shown that the concentration of the neurotoxic compound glutamate is highly variable between patients (33, 34). This finding suggests that the effect of glioblastomas on their surroundings, too, is variable. Third, glioblastomas harbor macrophages, microglia, and lymphocytes that secrete cytokines in addition to those secreted by the glioblastoma cells themselves (18, 25, 35–37). Thus, tumor-associated leukocytes probably contribute to the overall inflammatory effects of glioblastomas on the surrounding brain tissue.

The present study addresses two related issues: 1) whether glioblastomas entail inflammation that could mediate their destructive effect on the surrounding brain tissue, and 2) whether a destructive effect of glioblastomas on the surrounding brain tissue can be distinguished from displacement of brain tissue on pre-surgery MRI. With respect to the first issue, we analyzed cyst fluid from cystic glioblastomas and obtained quantitative data on cytokine concentrations, which we compared to cytokine concentrations in pus from bacterial brain abscesses, a highly pro-inflammatory environment. With respect to the second issue, we examined MRIs of the cystic and solid glioblastomas to evaluate whether displacement of brain tissue alone could account for the accommodation of the tumors within the restricted space of the skull, or whether destruction of brain tissue would be necessary to accommodate the tumor. We complemented these studies with an immunohistochemical analysis of cystic glioblastomas to look for evidence of a destructive effect of glioblastoma on brain tissue and to look for tumor-associated leukocytes that could shed light on the presence of cytokines in the cyst fluid of cystic glioblastomas.

## Methods

### Patients, neuroimaging, glioblastoma cyst fluid, and brain abscess pus

We prospectively enrolled patients with cystic glioblastoma and patients with bacterial brain abscess that underwent neurosurgery at The Department of Neurosurgery, The National Hospital, Oslo, Norway 2012-2021. All patients gave informed written consent to their participation. The study was approved by The Regional Committees for Medical and Health Research Ethics of Norway (concession# 2012/781 and 2012/617). There were no exclusion criteria. The glioblastoma cysts were identified as such intraoperatively from their highly fluid, non-solid, content. Cystic glioblastomas are rather infrequent, constituting approximately 8% of glioblastoma cases (28), hence the long period of enrolment.

To see if destruction of surrounding brain tissue could be a feature even of solid glioblastomas, we recruited 10 patients with solid glioblastoma retrospectively 2019-2021. Solid tumors, too, contained areas that could appear cystic or necrotic on MRI (compare **Figures 1**, **2**), but at surgery these areas were identified as solid and necrotic.

During tumor surgery, tumor cyst fluid was aspirated into a polypropylene syringe. The cyst fluid was rapidly centrifuged at 3000 g and 4°C for 10 minutes, and the supernatant was frozen at -80°C until analysis. Brain abscess patients underwent pus evacuation through a minimally invasive procedure as described (38). Pus was rapidly centrifuged at 3000 g, and the supernatant was frozen at -80°C until analysis.

The diagnosis of glioblastoma was based on histological examination. Isocitrate dehydrogenase (IDH) mutation status was available for all patients. Pus from brain abscess patients underwent bacterial identification with polymerase chain reaction (PCR) or culture methods as per hospital routine (39, 40).

For glioblastoma patients with cystic glioblastomas, number of days of corticosteroid treatment to reduce brain edema (41) was recorded together with blood leukocyte count on the day of neurosurgery.

### Cytokine measurements

The cytokines TNF-α, IFN-γ, IL-1β, IL-2, IL-4, IL-6, IL-8, IL-10, IL-12(p70), IL-13, MCP-1 (CCL2), the chemokine CXCL1 (Groα), and tissue factor were analyzed with the U-Plex Biomarker Group 1 (human) assay (Meso Scale Diagnostics, Rockville, MD, USA), which yields quantitative data on cytokine concentration in fluids. Simultaneously, some leukocyte markers were analyzed: soluble CD163 (sCD163), a marker of activated macrophages (42, 43), the soluble IL-2 receptor α-subunit, sCD25, a marker of lymphocytic activation (44–46), and the neutrophil marker myeloperoxidase (MPO; 47). IL-1β, IL-2, IL-12, TNF-α, and IFN-γ are considered typical Th1 cytokines, whereas IL-4, IL-10, and IL-13 are considered Th2 cytokines (48, 49). Tissue factor was analyzed as a pro-inflammatory and pro-coagulant factor (50) that is highly expressed in glioblastoma (51).

### MRI-based evaluation of displacement of brain tissue surrounding the glioblastomas

All but one glioblastoma patient (who had cystic glioblastoma) underwent pre-operative MRI, including T1-weighted magnetization–prepared, rapid gradient echo (MPRAGE) images before and after intravenous infusion of gadolinium-based contrast agent (Clariscan 279.3 mg/mL, 0.2 mL/kg bodyweight, GE Healthcare, USA) and T2-weighted images. MRI was repeated days to weeks after surgery. Tumor and cyst volumes were calculated semi-automatically from post contrast T1-weighted images with the Smartbrush program (Brainlab, Feldkirchen, Germany). This method provides a minimum estimation of tumor volume (4, 52). The cystic glioblastomas that underwent MRI were the same as those that were analyzed with respect to cytokine content.

Degree of tissue displacement (mass effect) caused by the tumors and cysts was evaluated by an experienced neuroradiologist (see Acknowledgements) and classified as minimal, moderate or pronounced based on the degree of midline shift and compression of the cerebral ventricles and subarachnoid spaces caused by the tumors (**Figure 2**). For correlation assessments, the rating of mass effect (minimal, moderate, pronounced) was converted to the values 1, 2, and 3, respectively. T2-weighted fluid-attenuated inversion recovery (FLAIR) signal was used to evaluate peri-tumoral edema, which was also graded as minimal, moderate, or pronounced (converted to 1, 2, and 3, respectively, according to 53). Hence, the evaluation of whether tumors had caused tissue destruction was based on a relative lack of mass effect in spite of a substantial tumor volume. Post-operative MRIs were included to evaluate the impression of a destructive effect of the tumors on the surrounding tissue, keeping in mind that tumor resection implies the removal of some of the brain tissue surrounding the tumor and that post-surgery radiation therapy may cause some degree of gliosis and tissue retraction. However, because the last author (DD) performed the surgeries, we know that the resection of surrounding brain tissue was limited.

### Histology

Tissue was removed from various parts of the glioblastomas during surgery, among them border zones between tumor and surrounding brain tissue, both white matter and neocortex. Tissue samples were fixed in 4% formaldehyde and embedded in paraffin. Paraffin sections were investigated with hematoxylin and eosin staining and with immunohistochemistry. For immunohistochemistry, we used antibodies against glial fibrillary acidic protein (GFAP), which stains glioblastoma cells (Glostrup, DK; product # M0761). Antibodies for neuronal markers were against neurofilament heavy chain (non-phosphorylated; Dako product # M0762), and the neuronal nuclear protein NeuN (Millipore-Chem; Merck, Darmstadt, Germany; product # MAB377, clone A60). For visualisation of macrophages and activated microglia we used antibodies against CD68 (DAKO product # M0814). For visualisation of T-lymphocytes we used antibodies against CD3 (Leica Biosystem, UK; product # CD3). For visualization of B-lymphocytes we used antibodies against CD20 (Dako product # M0755). Secondary antibodies were from rabbit (Dako). These antigens have been used previously as specific markers for glioblastoma-associated leukocytes (36).

### Data presentation and statistics

Data on cytokine concentrations and volumes of tumors, cysts, and brain abscesses are given as absolute values. Data were analyzed with respect to normality with the Kolmogorov-Smirnov test. Differences between cytokine levels in glioblastoma cyst fluid and brain abscess pus were analyzed with the Mann-Whitney U test or the Student’s *t*-test, as appropriate. Correlations were analyzed with Pearson’s or Spearman’s test, as appropriate. P-values < 0.05 were considered statistically significant.

## Results

### Patients, glioblastomas, and brain abscesses

Twenty-one patients with cystic glioblastoma, four women and 17 men, aged 26-78 years (median age 65 years) had tumors in any of the cerebral lobes. Three out of the 21 patients with cystic glioblastoma had tumors with IDH mutations; according to the recent classification system (2) these are WHO grade IV astrocytomas, but are included in this series as they were considered glioblastomas at the time of diagnosis and were macroscopically indistinguishable from the rest of the group. Ten patients with solid glioblastoma, five women and five men, aged 41-78 years (median 59 years), had tumors in any of the cerebral lobes; none of these tumors had IDH mutations.

The cyst fluid that was aspirated from the glioblastoma cysts was highly fluid, transparent and of a color that varied from faint yellowish to colorless. Centrifugation of cyst fluid samples (2-5 mL) caused the precipitation of a few cells that could be seen by light microscopy; these cells, which constituted a minimal fraction of the cyst fluid, were not characterized further. All but one of the glioblastoma patients received corticosteroid treatment for 1-83 days (median 14 days) to reduce peri-tumoral edema. Doses were initially 16 mg methylprednisolone four times per day; these were gradually reduced to 4 mg four times per day, the rate of dose reduction depending on the severity of symptoms and the clinical response to treatment. After tumor resection, all patients received adjuvant temozolomide treatment and radiation therapy (3).

Thirteen patients, three women and ten men, aged 24-72 years (median age 53 years) had bacterial brain abscesses in any of the cerebral lobes. Abscess volumes were 2.7-42 cm^3^ (median 21 cm^3^). The aspirated pus was highly viscous, opaque and of a color that varied from yellowish to brown. Microscopy prior to centrifugation showed a high density of leukocytes, mostly neutrophils, which constituted >50% of the pus volume. PCR or bacterial culture identified *Streptococcus intermedius* in eight patients*, Fusobacterium nucleatum* in three, *Aggregatibacter aphrophilus* in two, and *Parvimonas micra*, *Porphyromonas endodontalis*, *Propionebacterium acnes*, and β-hemolytic streptococci group G in one patient each. (In two pus samples, three bacterial species were identified: *F. nucleatum*, *P. micra*, and *P. acne*s in one; *S. intermedius*, *A. aphrophilus*, and *P. endodontalis* in another). The bacterial identity did not correlate with abscess volumes or cell marker or cytokine levels.

### Cell markers and cytokine levels in glioblastoma cyst fluid and brain abscess pus

**Figure 3:**
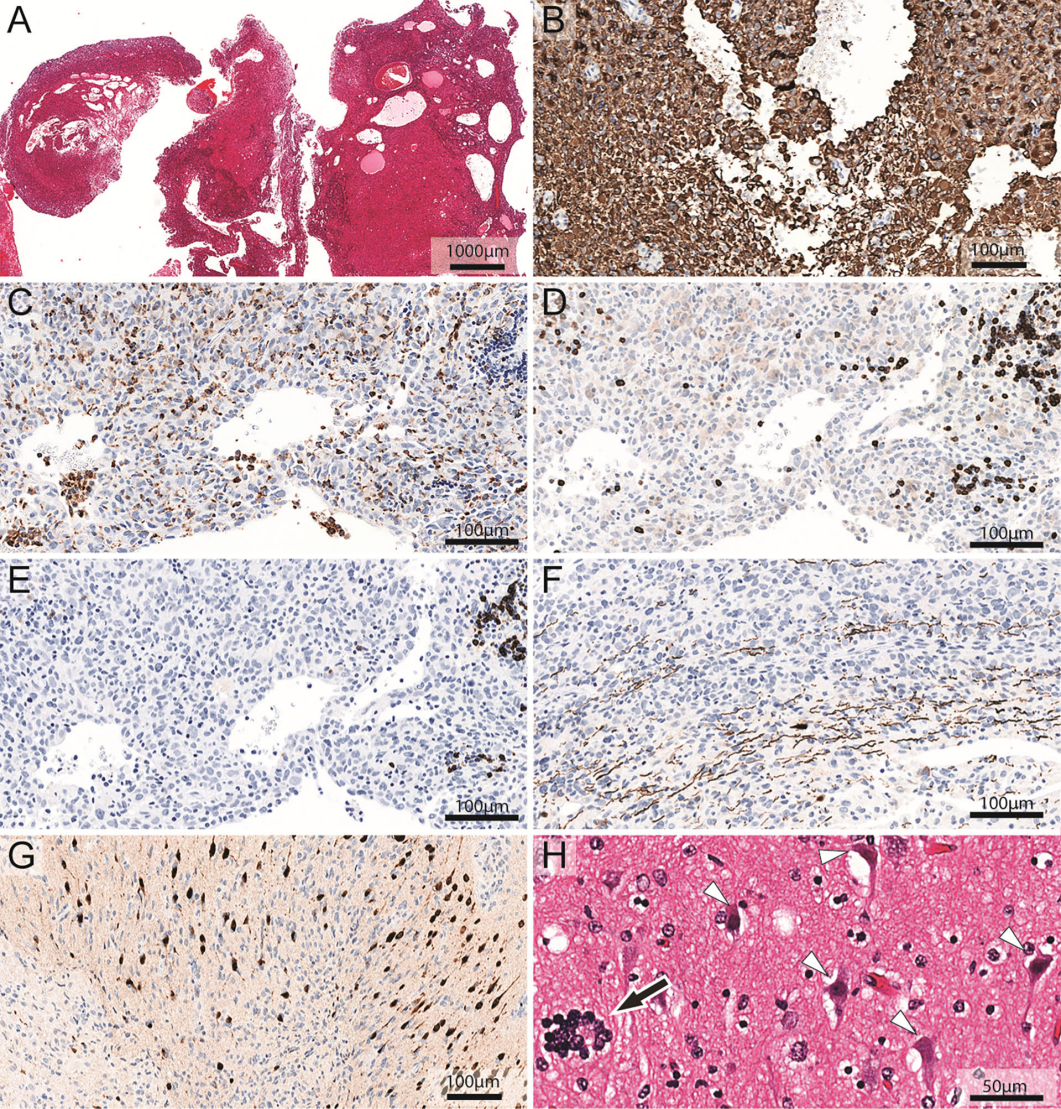
Neuropathological analysis of cystic glioblastoma. **(A)** Hematoxylin and eosin staining of a representative biopsy reveals intratumoral cysts. **(B)** Glioblastoma cells show strong immunoreactivity (brown color) for GFAP. **(C)** Anti-CD68 antibodies label macrophages and microglia cells (dark brown cells). The areas of the section without solid tissue represent cyst lumen. **(D)** Anti-CD3 antibodies label T-lymphocytes (dark brown cells). The areas of the section without solid tissue represent cyst lumen. **(E)** Anti-CD20 antibodies label immunoreactive B-lymphocytes (dark brown cells). The areas of the section without solid tissue represent cyst lumen. **(F)** The infiltrative growth pattern of the glioblastoma cells in white matter is evident among the axons that are stained dark brown for neurofilament heavy chain. **(G)** Glioblastoma cells infiltrate neocortical tissue where neurons are stained dark brown for NeuN. **(H)** Hematoxylin and eosin staining of neocortex shows damaged neurons (white arrowheads) with shrunken, triangular appearance and condensed chromatin. Prominent satellitosis (56) can be seen (black arrow). Please note the proximity of tumor-associated leukocytes to the cyst lumen **(C**–**E)**. Note also how macrophages and lymphocytes may both be scattered throughout the tumor and appear in groups **(C–E)** and how neurons (brown nuclei) embedded among tumor cells (blue nuclei) appear pycnotic in the center of **(G)**.

In glioblastoma cyst fluid, the concentration of sCD163, a marker of activated macrophages and microglia (42, 43) was significantly higher than in brain abscess pus (**Table 1**). This was true also for MCP-1, a pro-inflammatory chemokine that is secreted mostly by monocytes, macrophages, and microglia (54). The level of sCD25, a lymphocyte marker (44–46), was not significantly different in glioblastoma cyst fluid and brain abscess pus. In contrast, the concentration of MPO, which is released from neutrophils (47), was much higher in brain abscess pus, in keeping with pus being dominated by neutrophils and in agreement with a previous study showing that MPO is a dominant protein in brain abscess pus (38). Similarly, the concentration of IL-1β, which may be released from both neutrophils and macrophages (55), was much higher in brain abscess pus than in glioblastoma cyst fluid. Histological analysis of tissue from cystic glioblastomas showed the presence of macrophages and lymphocytes in glioblastoma and their proximity to the cyst fluid (**Figure 3**; se images C, D, and E, specifically).

**Table 1:** Cell markers and cytokines in glioblastoma cyst fluid and brain abscess pus.

		Glioblastoma (n=21)		Brain abscess (n=13)	
		Median	Min – Max	Median	Min – Max
**sCD163**	ng/mL	1874*	124 – 12281	**88**	6 – 2635
**sCD25**	pg/mL	**1587**	113 – 10910	**550**	232 – 9324
**MPO**	µg/mL	0.69***	0.03 - 29.2	**>180**	46 – >180
**MCP-1**	pg/mL	8898*	7 – 143652	**433**	72 – 38733
**TNF-α**	pg/mL	32*	n.d. – 1020	**62**	31 – 99
**IFN-γ**	pg/mL	7***	n.d. – 51	**48**	n.d. – 5947
**IL-1β**	pg/mL	13***	n.d. – 194	**9296**	103 – 19611
**IL-2**	pg/mL	5***	n.d. – 19	**24**	12 – 55
**IL-4**	pg/mL	1*	n.d. – 9	**4**	2 – 29
**IL-6**	pg/mL	**1064**	2 – 28921	**178**	66 – 32495
**IL-8**	pg/mL	**23585**	1 – 87416	**26272**	21153 – 27319
**IL-10**	pg/mL	4***	n.d. – 18	**11**	6 – 26
**IL-12**	pg/mL	3*	n.d. – 17	**8**	2 – 65
**IL-13**	pg/mL	37***	n.d. – 123	**102**	49 – 188
**CXCL1**	pg/mL	**639**	3 – 12701	**722**	33 – 13077
**TF**	pg/mL	**26**	1.1 – 508	**11**	0.4 – 114

Patients with cystic glioblastoma (n=21) or bacterial brain abscess (n=13) underwent neurosurgery with drainage of glioblastoma cyst fluid or brain abscess pus, which were analyzed for cytokines. sCD163 values are ng/mL, MPO values are µg/mL, the other values are pg/mL. Data are median, minimum, and maximum values. Asterisks: significantly different from corresponding values in brain abscess pus; *: p<0.05, **: p<0.01, ***: p<0.001 (Mann-Whitney U test or Student’s t-test, as appropriate). MPO levels exceeded maximum detectable value (180 µg/mL) even after 1:5 sample dilution, hence the use of “>180 µg/mL” in the table; this was the value recorded in 8 out of 13 patients. IFN, interferon, IL, interleukin; MCP, monocyte chemoattractant protein; MPO, myeloperoxidase; n.d., not detectable; TNF, tumor necrosis factor; TF, tissue factor. In calculating median values non-detectable levels were given a zero value. Bolds are correlation coefficients.

Cytokine levels were highly variable across the 21 glioblastoma cyst fluid samples (**Table 1**). For instance, levels of MCP-1, IL-6, IL-8, and the chemokine CXCL1 varied by >1000-fold. TNF-α levels varied similarly, from not being detectable in one patient to the maximum value being approximately 25 times higher than the median value. The glioblastoma cytokine levels correlated positively with one another (**Table 2**), reflecting that cytokine levels tended overall to be high, medium, or low.

**Table 2:** Correlations between levels of some cytokines and sCD25 in glioblastoma cyst fluid.

	IL-8	IL-6	TNF-α	CXCL1	sCD25
**MCP-1**	**0.76** p=5x10^-5^	**0.69** 0.00049	**0.56** p=0.0085	**0.62** p=0.0025	**0.70** p=0.00037
**IL-8**		**0.81** p=10^-5^	**0.68** p=0.00066	**0.71** p=0.00029	**0.63** p=0.00020
**IL-6**			**0.71** p=0.00033	**0.82** p<10^-10^	**0.61** p=0.0037
**TNF-α**				**0.85** p<10^-10^	**0.70** p=0.00044
**CXCL1**					**0.59** p=0.0048

Patients with cystic glioblastoma (n=21) underwent neurosurgery with drainage of cyst fluid. Data are Spearman’s correlation coefficients and corresponding p-values. The positive correlation indicates that inflammatory activity was overall high, medium or low. IL, interleukin; MCP, monocyte chemoattractant protein; TNF, tumor necrosis factor. Bolds are correlation coefficients.

In spite of the great variability in cytokine concentration, glioblastoma cyst fluid levels of TNF-α, IL-6, IL-8, and CXCL1 were not significantly different from those in brain abscess pus, and, as stated above, the level of MCP-1 was significantly higher in cyst fluid, all indicative of a highly pro-inflammatory glioblastoma environment (**Table 1**). The levels of IFN-γ, IL-1β, IL-2, IL-4, IL-10, IL-12, and IL-13, in contrast, were significantly higher in pus than in cyst fluid. The level of tissue factor, a non-cytokine pro-inflammatory and pro-coagulant compound, which may stimulate the release of cytokines such as IL-8 and the chemokine CXCL1 (50), was not significantly different in glioblastoma cyst fluid and brain abscess pus.

The three patients whose tumors harbored IDH mutations did not stand out in any way with respect to cyst fluid cytokine levels. For instance, their level of tissue factor, which reportedly is lower in tumors bearing IDH mutations (50), was 25, 46, and 89 pg/mL, respectively, which was at, or above, the median value for the group as a whole (**Table 1**).

The levels of several cytokines in glioblastoma cyst fluid correlated with blood leukocyte count (**Table 3**). The level of IL-10 in glioblastoma cyst fluid correlated with the number of days on steroid treatment (r=0.66; p=0.0015) in line with previous studies (57, 58); for the other cytokines, including the other Th2 type cytokines IL-4 and IL-13 (see Methods, section Cytokine measurements), there was no correlation with number of days of corticosteroid treatment (r values from -0.27 to 0.05; p values 0.23-0.80). Similarly, there was no correlation between number of days of corticosteroid treatment and blood leukocyte levels (r= -0.11; p=0.6). It should be kept in mind, however, that the dose of methylprednisolone was not the same throughout the treatment period (see Section Results, Patients, glioblastomas, and brain abscesses), which could obscure correlations with corticosteroid treatment.

**Table 3:** Correlations between blood leukocyte count and levels of cell markers and cytokines in glioblastoma cyst fluid.

sCD163	sCD25	MPO	MCP-1	TNF-α	IFN-γ	IL-1β	IL-2
**0.40**	**0.52** p=0.017	**0.56** p=0.013	**0.44** p=0.049	**0.62** 0.0027	**0.47** p=0.029	**0.43** p=0.0052	**0.51** p=0.019
**IL-4**	**IL-6**	**IL-8**	**IL-10**	**IL-12**	**IL-13**	**CXCL1**	**TF**
**0.37**	**0.43**	**0.61** p=0.0031	**0.44** p=0.044	**0.38**	**0.38**	**0.63** p=0.0025	**-0.001**

Patients with cystic glioblastoma (n=21) underwent neurosurgery with drainage of cyst fluid. Data are correlation coefficients (Pearson’s or Spearman’s, as appropriate) and corresponding p-values below 0.05. IFN, interferon; IL, interleukin; MCP, monocyte chemoattractant protein; MPO, myeloperoxidase; TNF, tumor necrosis factor; TF, tissue factor. Bolds are median values.

Median survival for the whole patient group was 18.5 months (range 2-100). Two patients, whose tumors carried IDH mutations survived for 81 and 100 months; one patient, whose glioblastoma did not carry an IDH mutation, survived for 85 months. When patients carrying IDH mutation were excluded, median survival was 17.5 months (range 2-85). There were no correlations between the level of any cytokine and survival, whether or not patients whose tumors carried IDH mutations were excluded (r= -0.22 – 0.28; p>0.2).

### Glioblastoma volumes and assessment of displacement (mass effect) *vs*. destruction of surrounding brain tissue

Pre-operative MRIs were available for 20 out of the 21 patients with cystic glioblastomas that were analyzed with respect to cyst fluid cytokine content (the last patient underwent pre-operative CT only). Cyst volumes were 5-60 cm^3^ (median 28 cm^3^), whereas the volume of tumor tissue was 3.4-78 cm^3^ (median 19 cm^3^). The cyst volumes constituted 16-83% of the total tumor (solid tumor + cyst) with a median value of 48%.

**Figure 4:**
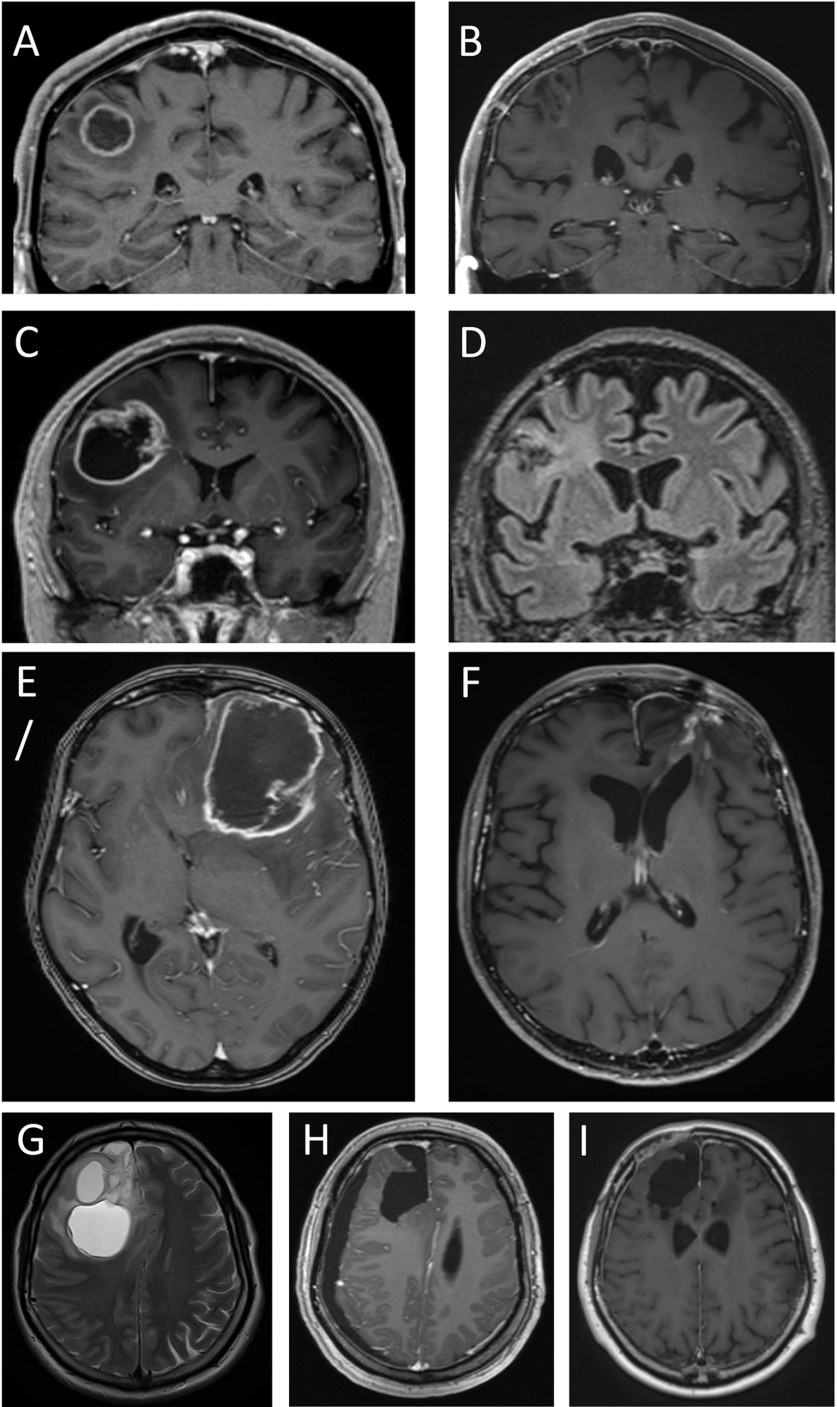
Four cystic glioblastomas before and after tumor surgery. T1 weighted MPRAGE MRIs after intravenous injection of gadolinium-based contrast medium (which gives the ring-formed bright signal in most images), except in D and G, which are T2-weighted and T2-weighted FLAIR, respectively. Images **(A, C, E, G)** show pre-surgical MRIs. Note the modest displacement of surrounding brain tissue (compare with contralateral side). Images **(B, D, F, H, I)** show MRIs after 240, 295, 330, 39 days, and 45 months, respectively. Tumors in images **(A**, **C)** were assessed as having minimal mass effect, tumors in images **(E**, **G)** had pronounced mass effect. Note the absence of brain tissue where the tumor and cyst resided; this comprises white matter and adjacent neocortex.

In the patients with cystic glioblastomas, mass effect correlated with cyst volume (r=0.59; p=0.0079) and with tumor volume (r=0.47; p=0.045). In three of the 20 patients, mass effect was deemed minimal, in eight it was moderate, and in nine it was pronounced. In the patients with minimal and moderate mass effect, the relatively low degree of tissue displacement, in spite of substantial tumor and cyst volumes, was consistent with some brain tissue having been destroyed by tumor growth (**Figure 4**). In some patients, peri-tumoral edema contributed visibly to whatever mass effect there was (**Figures 4C, E**), but peri-tumoral edema and mass effect were not correlated in the group as a whole (r=0.15; p=0.55). Involvement of the neocortex could be seen in some patients with cystic glioblastomas (**Figures 4C, E, G**); this was also seen histologically (**Figures 3G, H**).

There were no significant correlations between mass effect on the one hand and cyst fluid levels of leukocyte markers or cytokines on the other hand (r values from -0.35 to -0.02; p values 0.15-0.95). Similarly, there were no significant correlations between degree of peri-tumoral edema as seen on T2-weighted FLAIR MRI on the one hand and levels of leukocyte markers or cytokines on the other (r values from -0.16 to 0.05; p values 0.52-0.98).

**Figure 5:**
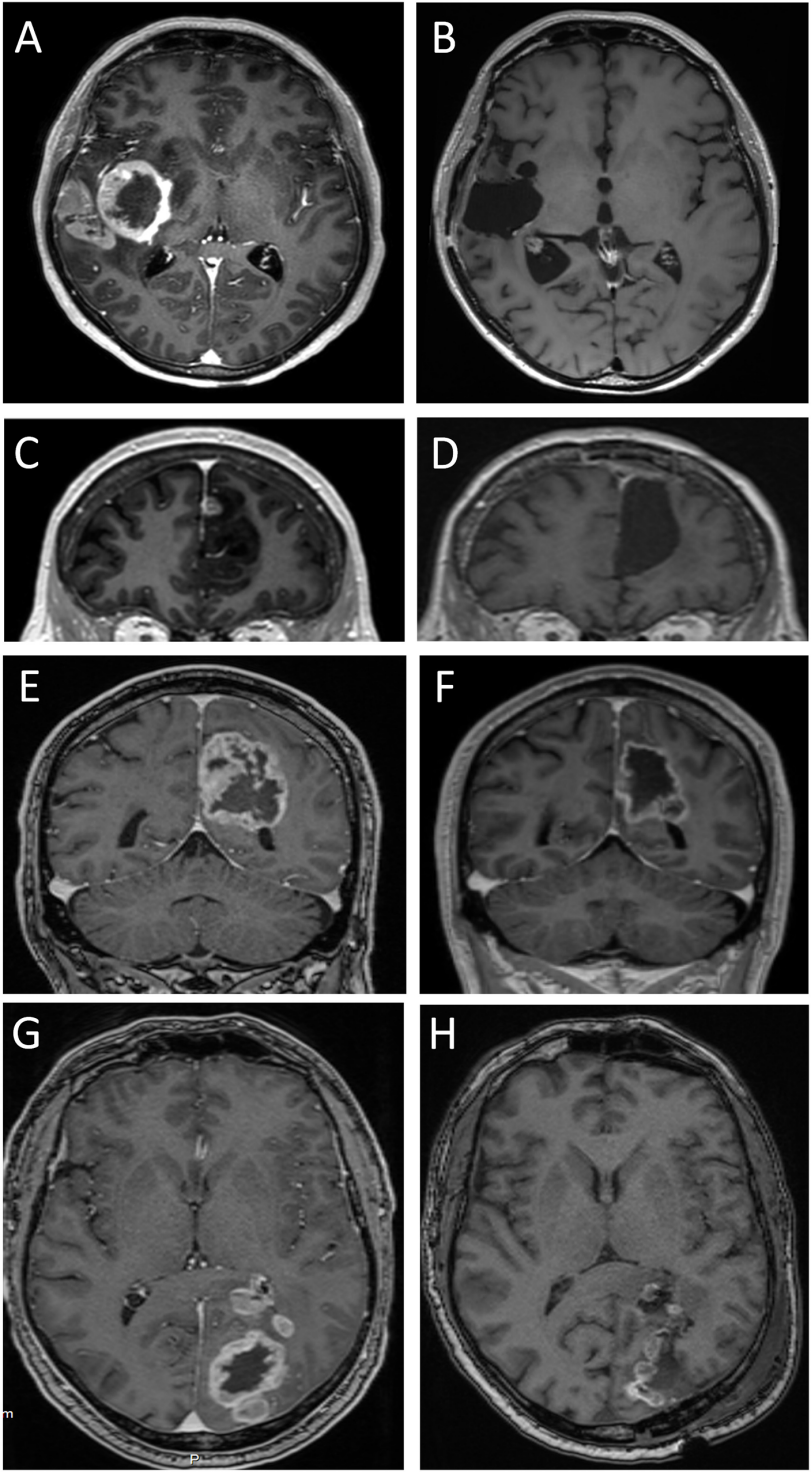
Four solid glioblastomas before and after tumor surgery. T1-weighted MPRAGE MRIs were obtained after intravenous injection of gadolinium-based contrast medium (which gives a bright, ring-formed signal in most images). Images **(A, C, E, G)** show pre-surgical MRIs. Tumors in images **(A**, **C)** were assessed as having minimal mass effect. Note the modest displacement of surrounding brain tissue (compare with contralateral side). Tumors in images **(E**, **G)** were assessed as having moderate and pronounced mass effect, respectively. Images **(B, D, F, H)** show MRIs after 400, 167, 26, and 4 days, respectively. Note the absence of brain tissue where the tumor resided. This includes both white matter and the overlying neocortex.

The contrast-enhancing component of solid glioblastomas (n=10) measured 1-58 cm^3^ (median volume 16 cm^3^). Displacement of brain tissue (mass effect) correlated with tumor volume (r=0.65; p=0.041). However, in five of the 10 patients, mass effect of the tumor was deemed minimal, in four it was moderate, and in one it was pronounced. In some patients with minimal mass effect, the relative absence of displacement of brain tissue in spite of a substantial tumor size appeared consistent with the notion that some brain tissue had been destroyed due to tumor growth (**Figure 5**). In some patients, the involvement of the neocortex, whether over the brain convexities (**Figures 5A, G**) or parasagittally (**Figures 5C, E**), was apparent on MRI.

Histological analysis of cystic glioblastomas showed tumor cell infiltration of white matter and neocortex (**Figures 3F, G**). Neocortical neurons appeared damaged (**Figure 3H**). Although these histological images do not prove the neurotoxicity of glioblastoma cells in human brain, they illustrate the proximity of tumor cells to vulnerable neurons.

### Assessment of post-surgery MRIs in glioblastoma patients

MRI performed days to weeks after surgical removal of the glioblastomas showed the presence of a resection cavity in patients with cystic or solid glioblastomas (**Figures 4H, I**; **Figures 5B, D, F, H**). In some patients, the loss of neocortical tissue was evident after tumor resection, whether over the brain convexities (**Figures 4B, D, F, H, I**; **Figures 5B, H**), or parasagittally (**Figures 4H, I**; **Figures 5D, F**). In some patients, loss of brain tissue was seen as tissue retraction leading to enlargement of the lateral ventricles (**Figure 4F**). These observations were a further suggestion that some brain tissue had been destroyed by the glioblastomas.

## Discussion

### High, but variable concentrations of pro-inflammatory cytokines in the glioblastoma environment. Possible impact on the surrounding brain tissue

We report here on concentrations of pro-inflammatory cytokines as they would appear in the microenvironment of cystic glioblastomas and the surrounding brain tissue. In general, cytokine levels were high. The median concentrations of TNF-α, IL-6, IL-8, MCP-1, and the chemokine CXCL1 in glioblastoma cyst fluid were far higher than previously published serum values for SARS covid-19 patients, patients with sepsis, and healthy controls (59–66). The values reported here are similar to CSF values after intracerebral and intraventricular hemorrhage, an acutely inflammatory condition (67). The conclusion that pro-inflammatory markers are high in the glioblastoma microenvironment was corroborated by the comparison with pus from bacterial brain abscesses, a highly pro-inflammatory environment: glioblastoma cyst fluid had levels of TNFα, IL-6, MCP-1, CXCL1, sCD163, sCD25, and tissue factor that were similar to or higher than those in pus, although the level of several other cytokines was higher in pus. The levels of MCP-1, IL6 and IL-8 in glioblastoma cyst fluid in the present study were similar to values reported previously in glioblastoma cyst fluid (**Table 4**); as can be seen from the table, some other inflammation-related proteins have also been detected in glioblastoma cyst fluid. However, in spite of the usefulness of analyzing glioblastoma cyst fluid for quantitative data on cytokines and related proteins, relatively few studies have been performed on this fluid for the last 30 years.

**Table 4:** Inflammation-related proteins identified in cyst fluid from cystic glioblastomas, as reported in the literature.

Pro-inflammatory proteins in cyst fluid	Concentration in cyst fluid	Method	Authors and publication dates
TGF-β2	n.d.	SDS-PAGE	Bodmer et al., 1991 (68)
IL-8	612-7,787 pg/mL	ELISA	Van Meir et al., 1992 (69)
MCP-1	2,400-15,000 pg/mL	ELISA	Kuratsu et al., 1993 (70)
VEGF	2,252- 1263,000 pg/mL	ELISA	Takano et al., 1996 (71); Stockhammer et al., 2000 (72)
Tenascin-C	150-1,368 ng/mL	SDS-PAGE	Jallo et al., 1997 (73)
MIP-1β	0-50 pg/mL	ELISA	Ishii et al., 1998 (74)
Ferritin, basigin, TNF	n.d.	SELDI-TOF	Hoelscher et al., 2013 (75)
IL-6	Approx. 500 pg/mL	ELISA	Shen et al., 2014 (76)
L1CAM	6,118 ± 4,095 ng/mL	ELISA	Wachowiak et al., 2018 (77)
Bradykinin, TREM2, ALCAM, and more	n.d.	LC-MS/MS	Dahlberg et al., 2022 (24)

The table shows, in chronological order, various inflammation-related proteins that have been identified in glioblastoma cyst fluid, their concentrations, the method used for detection, and the year of publication. Please note that in the Kuratsu etal. (70) paper, cyst fluid was from patients with anaplastic astrocytoma. The review by Shen etal. (76) includes data from 8 studies published 2003-2013. The number of cyst fluids analyzed in each study varied base on 1 to 25.

ALCAM, Activated leukocyte cell adhesion molecule; CAM, Cell adhesion molecule; ELISA, Enzyme-linked immunosorbent assay; IL, Interleukin; LC, Liquid chromatography; MCP, Monocyte chemoattractant protein; MIP, Macrophage inflammatory protein; MS, Mass spectrometry; n.d., Not determined; SELDI-TOF, Surface-enhanced laser desorption ionization time of flight; SDS-PAGE, Sodium dodecyl sulfate–polyacrylamide gel electrophoresis; TGF, Transforming growth factor; TNF, Tumor necrosis factor; TREM, Triggering receptor expressed on myeloid cells; VEGF, Vascular endothelial growth factor.

The high level of TNF-α in brain abscess pus was similar to that reported previously by Bajpai et al. (78). The importance of TNF-α, IL-6, and other cytokines for the acute host response against severe bacterial brain infection has been documented in experimental studies (79–81). The high concentration of these cytokines in glioblastoma cyst fluid is all the more remarkable.

We found that the levels of several cytokines in glioblastoma cyst fluid correlated with blood leukocyte counts. At present, we cannot say whether this correlation reflected systemic effects of the glioblastomas or, vice versa, that a high blood level of leukocytes leads to a high number of leukocytes entering the glioblastomas with ensuing high levels of cytokines. In either case, this observation may point to an important interaction between glioblastomas and the circulation.

A purpose of the present study was to investigate whether an inflammatory effect of glioblastomas could contribute to a destructive effect on the surrounding brain tissue. Indeed, the concentrations of inflammatory markers in glioblastoma cyst fluid pointed to a highly pro-inflammatory and potentially destructive environment. However, the concentrations of cytokines needed to affect brain tissue adversely is currently not known. Most cytokines have been reported to be involved in both neurodegenerative and regenerative processes. IL-8 receptors have been identified on axons in human brain (82), and the CXCL1 receptor, CXCR2, is expressed in CNS oligodendrocytes (83), which would allow for an inflammatory response in white matter to the high levels of IL-8 and CXCL1 in glioblastoma cyst fluid. However, CXCL1, which is formed by glioblastoma cells, leukocytes and astrocytes alike, and which could be important for tumor immunogenicity (84), may also have neuroprotective effects (85, 86). Overexpression of IL-6 in the brain causes neurodegeneration (87), but other studies have suggested a physiological role for IL-6 in the stabilisation of CNS axonal microtubules (88). Similarly, TNF-α may cause neuronal and oligodendroglial dysfunction and death, while at the same time acting as a trophic factor for brain cells (89), and the pro-inflammatory cytokines IL-6 and MCP-1 have roles in axonal regeneration after mechanical damage to the spinal cord (90–93). Therefore, we are not able to delineate the exact effects of the various cytokines, or their combined action, on the brain tissue surrounding glioblastomas. More research is needed on the effects of cytokines on the function, survival, and degeneration of the brain tissue surrounding a glioblastoma. There was, however, a striking variability in cytokine levels among patients. Thus, it may be assumed that the degree of inflammation probably varies between glioblastomas and that inflammation, to the extent that it affects normal brain tissue or tumor growth (25, 35, 37), does so differentially.

Some of the proteins detected in this study are considered to be fairly cell-specific: sCD163 and MCP-1 are markers of activated macrophages and microglia (42, 43, 54), sCD25 is a marker of activated lymphocytes (44–46), and MPO is primarily a marker of neutrophils (47). The high levels of these proteins suggested the presence of the corresponding leukocytes in glioblastomas or their cysts. Histologic analysis confirmed the presence of macrophages and/or microglia and lymphocytes in cystic glioblastomas, in agreement with previous studies in solid glioblastomas (18, 36).

The degree of edema surrounding a glioblastoma has been proposed as a predictor of poor survival (94, 95). The edema has been ascribed to the production of vascular endothelial growth factor (VEGF) by glioblastomas (96). In the present study, the high concentrations of various cytokines in the glioblastoma environment likely contributed to capillary leakage and edema formation. For instance, TNF-α, IL-1β, IL-6, and IL-8 have all been shown or suggested to mediate brain edema formation independently of one another (97–101).

### The glioblastoma-mediated destruction of the surrounding brain tissue may be assessed qualitatively on pre-surgery MRI

MRI observations in the present study complement previous studies on the destructive effect of glioblastoma on the surrounding brain tissue. This destructive effect has been documented by magnetic resonance spectroscopy and diffusion tensor imaging of the brain (10, 11), by the high circulating levels of neurofilament in glioblastoma patients (12) and in experimental studies (14–16). In the present study, a destructive effect of glioblastoma was evident, because displacement alone (mass effect) could not account for the accommodation of the tumor within the brain tissue. This observations points to the need for neuroprotective strategies in glioblastoma therapy. Previously, glutamate receptor blocker perampanel has received interest as a drug that could reduce tumor growth and at the same time act as a neuroprotective agent, as has been shown in experimental animal studies (102), but the possibility of neuroprotection through antiepileptic drug treatment has been questioned (103). The finding in the present study of very high concentrations of cytokines in the cyst fluid of cystic glioblastomas points to immunomodulation as another possible neuroprotective approach.

Our assessment of the destruction caused by glioblastoma was qualitative. Future studies are needed to develop a quantitative method for MRI-based assessment of glioblastoma’s destructive effect in human brain.

The destructive process seemed to affect both white matter and the overlying neocortex. Several mechanisms could underlie a destructive process in the brain tissue surrounding the glioblastoma, but, presumably, these mechanisms are somewhat different for grey and white structures. A high inflammatory activity could be destructive in both types of brain tissue, whereas glutamate, which may be released by glioblastomas (15, 19, 20), would probably affect cortical neurons with glutamate receptors more than white matter. In contrast, physical strain, including stretching and distortion of brain tissue, could have a more pronounced effect on white matter axons. All destructive mechanisms are likely to produce secondary inflammatory responses that could be reflected in the cyst fluid levels of cytokines.

We did not see any correlation between cyst fluid levels of cytokines on the one hand and MRI-based assessment of displacement of brain tissue or peri-tumoral edema on the other. It remains to be investigated, with a method for quantitative assessment of brain tissue destruction, whether glioblastoma cytokine concentrations correlate with destruction of brain tissue.

### Limitations

There are limitations to the comparison of glioblastoma cyst fluid to brain abscess pus with respect to cytokine levels. Even though brain abscess pus is a highly pro-inflammatory environment with a high content of neutrophils, myeloperoxidase, and various cytokines (38, 78), it is also a hostile environment with low concentration of glucose, low pH, and high ammonia levels (39, 104). These conditions may be unfavorable for cytokine formation. Further, differences in cytokine levels between glioblastoma cyst fluid and brain abscess pus may reflect differences in cell types and cell density in the two fluids (34, 104). However, in spite of these limitations, it appears clear that the level of pro-inflammatory factors is high in both fluids.

We did not characterize our tumor samples with respect to genetic alterations that are common in glioblastomas: TERT promoter mutation, EGFR amplification, or gain of chromosome 7 combined with loss of chromosome 10 (2), and so we do not know whether these genetic alterations would influence the inflammatory environment of glioblastoma.

Most glioblastomas are not cystic (28), and we cannot say whether the concentrations of cytokines in glioblastoma cyst fluid would be similar in solid tumors. Cytokines are assumed to contribute to the growth of solid glioblastomas (18, 25, 35, 37), but their concentration in the extracellular fluid of solid tumors remains a topic for further research.

The assessment of the destruction of brain tissue caused by glioblastoma was done in a qualitative manner by an experienced neuroradiologist. We have not presented a method for quantitative determination of destruction of the brain tissue surrounding a glioblastoma. In the absence of a quantitative method, we were unable to look for correlations between cyst fluid cytokine levels and degree of destruction of brain tissue. Such a method could be a valuable tool for prognostic evaluation of glioblastoma surgery, as displaced brain tissue would presumably regain some function after surgery, whereas destroyed tissue would not.

We did not follow our patients with MRIs to see if cytokine levels in glioblastoma cyst fluid correlated with formation of drop metastases or with spread of the tumor within the brain tissue. Cytokines probably have an important role in the proliferation and migration of glioblastomas (17, 18). The lack of a correlation between the levels of the measured cytokines on the one hand and patient survival on the other could suggest that any correlation between cytokine levels and glioblastoma migration and metastasis relies on cytokines other than the ones investigated in the present study.

We have not presented data on the expression of cytokine receptors in the brain tissue surrounding the glioblastomas. Expression of cytokine receptors would be a prerequisite for glioblastoma-derived cytokines to cause tissue damage. Investigation of cytokine receptor expression in the peri-tumoral brain tissue remains a task for future histopathological research.

## Data availability statement

Restrictions apply to the datasets: The datasets presented in this article are not readily available due to issues of privacy, some raw data may not be made available upon request. Requests should be directed to the corresponding author(s).

## Ethics statement

This study was reviewed and approved by The Regional Committees for Medical and Health Research Ethics of Norway (concession# 2012/781 and 2012/617). The patients/participants provided their written informed consent to participate in this study.

## Author contributions

DD provided biological material and contributed to data analysis and drafting of the manuscript. PN provided histological analyses and contributed to data analysis and drafting of the manuscript. BeH provided cytokine analysis and contributed to data analysis and drafting of the manuscript. BjH contributed to data analysis and drafted the manuscript. All authors contributed to the article and approved the submitted version.

## Acknowledgments

The authors wish to thank Ellen Lund Sagen for technical assistance with cytokine measurements and Dr. Anna Latysheva, PhD, for expert neuroradiological evaluation of MRIs.

## Conflict of interest

The authors declare that the research was conducted in the absence of any commercial or financial relationships that could be construed as a potential conflict of interest.

## Publisher’s note

All claims expressed in this article are solely those of the authors and do not necessarily represent those of their affiliated organizations, or those of the publisher, the editors and the reviewers. Any product that may be evaluated in this article, or claim that may be made by its manufacturer, is not guaranteed or endorsed by the publisher.
